# A Treatise on Epidemic Cholera

**Published:** 1855-09

**Authors:** 


*A Treatise on Epidemic Cholera.* By HORATIO GATES JAMESON, Sr., M. D., Member of the Medical and Chirurgical Faculty of Maryland; Professor of Surgery; Member of the Philosophical Societies of Berlin and Moscow, &c. Philadelphia: Lindsay & Blakiston.

No subject can ever become old, in the sense of wearisome, in science, except to the mere *dilettante.* Until we have classified all the facts, and solved the mysteries, of a medical topic, let us not cease to welcome new and intelligent observations, or even ratiocinations upon it. This work contains much of both. Nor is it at all a mere compilation—Dr. Jameson having been a veteran in the practice as well as in the study of his profession. In 1832, as Consulting Physician to the City of Baltimore; and subsequently, in York and Columbia, Pa., and elsewhere, he has seen and treated a large number of cases of the disease of which he writes. He has travelled, in fact, in pursuit of data in regard to it, with a zeal akin to that which made the late Dr. Drake so great a light in American inductive medicine. The reader upon Cholera will find in the work of Dr. J. all the leading facts in regard to the natural history of the disease,—in its forms of *cholerine*, *cholero-dysentery*, and *cholera lethalis.* The style is easy, agreeable and vigorous; so that it makes not only a useful, but a very *readable* book. The doctrine of the non-contagiousness of the disease, and of the inefficacy of quarantine or other seclusive measures, is emphatically and very forcibly set forth. Other noticeable features of the Treatise are, the favor with which bloodletting in the early stage of cholera is spoken of, on the ground of actual experience; some extremely sensible remarks upon injection or transfusion, as a remedy; and the mention of the use of lard as an external application in the cold stage. The scope of the work has allowed the author to introduce a number of extracts from other treatises and periodicals, some of which are of great value; as, for instance, the account of the "physiolo-surgical" experiments of Dieffenbach in Berlin; the excellent Notification of the General Board of Health of England in 1853; and the summary of observations by Schönbein, Bœckel and others upon ozone. We regret that opportunity was not afforded, also, of noticing the experiments and observations upon this subject of Dr. Clemens, of Frankfort-on-the-Maine: which go far towards establishing a more consistent theory of the relations of ozonometry to hygiene, than has yet been suggested.

Curious facts are related, as having occurred in the vicinity of Baltimore, which appear to support, to a certain extent, the opinion of Dr. Bury, that *copper-works* have some preservative influence against cholera.

The view of Dr. Jameson is, that a *binary* causation is essential to the development of a cholera epidemic; its elements being, 1st, a general or ubiquitous *morbific cycle*, and 2d, *local malaria;* to which are often added, also, *fortuitous* causes,—in the imprudence or other accidental circumstances of individuals.

Although it has less of method in the arrangement, and more diffuseness and repetition, than will be agreeable to many readers, we believe that this treatise of Dr. Jameson will be found to be a valuable addition to the most select medical library, and an appropriate monument of the devotion, in advanced years, of one who has served through a long career as a votary of medical science. H.

